# Methyltransferase-like 3 Modulates Severe Acute Respiratory Syndrome Coronavirus-2 RNA N6-Methyladenosine Modification and Replication

**DOI:** 10.1128/mBio.01067-21

**Published:** 2021-07-06

**Authors:** Xueyan Zhang, Haojie Hao, Li Ma, Yecheng Zhang, Xiao Hu, Zhen Chen, Di Liu, Jianhui Yuan, Zhangli Hu, Wuxiang Guan

**Affiliations:** a Center for Emerging Infectious Diseases, Wuhan Institute of Virology, Center for Biosafety Mega-Science, Chinese Academy of Sciences, Wuhan, Hubei, China; b University of Chinese Academy of Sciences, Beijing, China; c College of Life Sciences and Oceanography, Shenzhen University, Shenzhen, China; d College of Physics and Optoelectronic Engineering, Shenzhen University, Shenzhen, China; e Hanshan Normal University, Chaozhou, China; f Nanshan District Center for Disease Control and Prevention, Shenzhen, China; Virginia Polytechnic Institute and State University

**Keywords:** methyltransferase-like 3, respiratory syndrome coronavirus-2, N6-methyladenosine, viral replication

## Abstract

The coronavirus disease 2019 pandemic caused by severe acute respiratory syndrome coronavirus-2 (SARS-CoV-2) is an ongoing global public crisis. Although viral RNA modification has been reported based on the transcriptome architecture, the types and functions of RNA modification are still unknown. In this study, we evaluated the roles of RNA N6-methyladenosine (m^6^A) modification in SARS-CoV-2. Our methylated RNA immunoprecipitation sequencing (MeRIP-Seq) and Nanopore direct RNA sequencing (DRS) analysis showed that SARS-CoV-2 RNA contained m^6^A modification. Moreover, SARS-CoV-2 infection not only increased the expression of methyltransferase-like 3 (METTL3) but also altered its distribution. Modification of METTL3 expression by short hairpin RNA or plasmid transfection for knockdown or overexpression, respectively, affected viral replication. Furthermore, the viral key protein RdRp interacted with METTL3, and METTL3 was distributed in both the nucleus and cytoplasm in the presence of RdRp. RdRp appeared to modulate the sumoylation and ubiquitination of METTL3 via an unknown mechanism. Taken together, our findings demonstrated that the host m^6^A modification complex interacted with viral proteins to modulate SARS-CoV-2 replication.

## INTRODUCTION

The coronavirus disease 2019 (COVID-19) pandemic is caused by severe acute respiratory syndrome coronavirus-2 (SARS-CoV-2), which belongs to the genus *Betacoronavirus* in the *Coronavirinae* subfamily of the *Coronaviridae* family (1–3). The rapid transmission of COVID-19 has been a major global challenge. Similar to the other two β-category coronaviruses, SARS-CoV-2 harbors a positive-sense, single-stranded RNA genome of approximately 30 kb, with 80% and 50% homology to SARS-CoV and Middle East respiratory syndrome coronavirus (MERS-CoV), respectively (4).

Internal chemical modifications of viral RNA play key roles in the regulation of viral infection. N6-methyladenosine (m^6^A), 5-methylcytosine (m^5^C), and N4-acetylcytidine (ac4C) have been reported to be involved in the viral life cycle (5–10). m^6^A is one of the most abundant internal RNA modifications (11, 12). The m^6^A machinery consists of “writers,” “erasers,” and “readers.” The writers, including methyltransferase-like (METTL) 3, METTL14, WT1-associated protein (WTAP), and other proteins, catalyze the transfer of the m^6^A modification (13–23). The erasers fat mass and obesity-associated protein (FTO) and AlkB homolog 5 (ALKBH5) are m^6^A demethylases that remove the methyl groups from RNA (22–25). The readers contain a YT521-B homology (YTH) motif that binds to m^6^A sites and play critical roles in mRNA stability (26–28), RNA processing (25), RNA structure (29), and translation (30, 31).

The internal m^6^A modification of viral RNA was identified in viruses that replicate in the cytoplasm, such as vesicular stomatitis virus, vaccinia virus, and reovirus, 40 years ago (32–36). However, the function of m^6^A was only recent elucidated in hepatitis C virus (HCV), Zika virus (ZIKV), dengue virus, yellow fever virus, and West Nile virus (37, 38). In viruses that replicate in the nucleus, such as human immunodeficiency virus (HIV), simian virus 40, Kaposi’s sarcoma-associated herpesvirus, and influenza virus, viral m^6^A modifications have been shown to affect viral replication and gene expression (39–46). Recent studies have found that m^6^A is present on SARS virus RNA and affects virus replication (47, 48), but the specific molecular mechanism of m^6^A regulating virus replication is still unclear. At least 41 sites modified on the SARS-CoV-2 genome are potential sites of RNA modification, which is particularly enriched at genomic nucleotide positions 28500 to 29500 (49).

Accordingly, in the current study, we investigated the presence of the roles of m^6^A modification in SARS-CoV-2 RNA using methylated RNA immunoprecipitation sequencing (MeRIP-Seq) and Nanopore direct RNA sequencing (DRS). Overall, our findings demonstrated that the host m^6^A modification complex interacted with viral proteins and modulated SARS-CoV-2 replication.

## RESULTS

### SARS-CoV-2 infection altered the expression patterns of m^6^A methyltransferases and demethylases.

**FIG 1:**
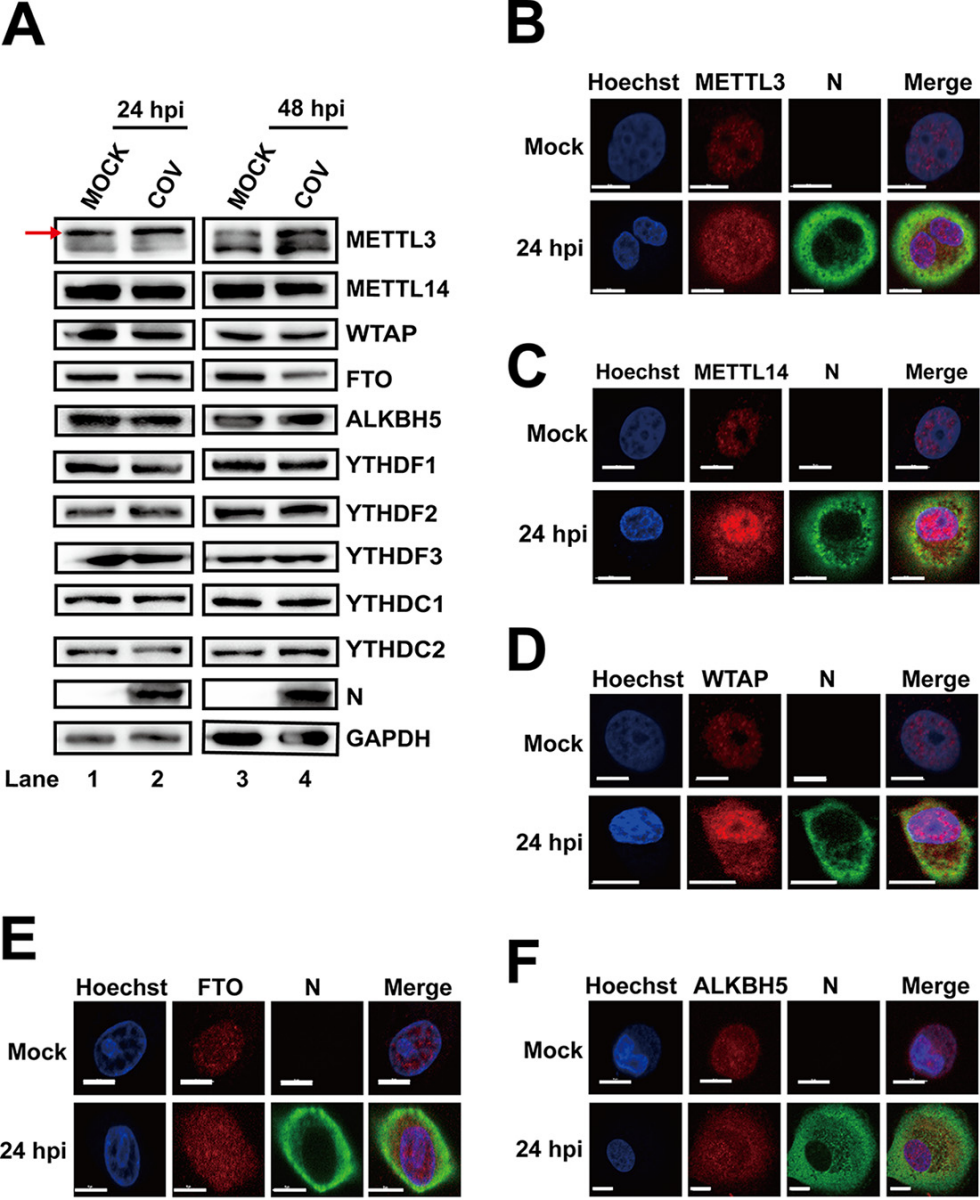
SARS-CoV-2 infection influenced the expression patterns of m^6^A-related proteins. (A) Western blotting. Vero E6 cells infected with SARS-CoV-2 (MOI = 0.01) were harvested at 24 and 48 hpi. Western blotting was performed with antibodies as indicated. GAPDH was used as a loading control. The arrow represents the METTL3-specific band. (B to F) Confocal microscopy images of SARS-CoV-2- or mock-infected Vero E6 cells. The nucleus (blue) and virus protein N (green) were labeled with Hoechst and anti-N-specific antibodies, respectively. The methyltransferases and demethylases were stained with antibodies as indicated. Scale bars, 5 μm.

The m^6^A methyltransferases and demethylases are mainly localized in the nucleus. Infection by viruses that replicate in the cytoplasm, such as enterovirus 71 (EV71), HCV, ZIKV, and porcine epidemic diarrhea virus (PEDV), affects the expression and localization of methyltransferases and demethylases to facilitate their RNA m^6^A modifications, which influences viral replication (37, 38, 50–52). To check whether SARS-CoV-2 infection had a similar effect, SARS-CoV-2-infected Vero E6 cells were harvested. The expression of viral N and m^6^A machinery proteins was assessed by Western blotting with corresponding antibodies (Fig. 1A). Our results showed that the expression of METTL3 was increased at 48 h postinfection (hpi), whereas the expression levels of METTL14 and WTAP were not affected (Fig. 1A). The expression of the demethylase FTO decreased at 48 hpi, whereas that of ALKBH5 was not changed after infection. Moreover, the expression of the m^6^A binding proteins YTHDF1 to -3, YTHDC1, and YTHDC2 was not altered during SARS-CoV-2 infection (Fig. 1A).

Previous studies have shown that m^6^A methyltransferases and demethylases colocalize with nuclear speckle markers and that viral infection affects the subcellular localization of m^6^A-related proteins. Because SARS-CoV-2 infection affects the expression of METTL3 and FTO, we next determined the effects of SARS-CoV-2 infection on the localization of methyltransferases and demethylases. Consistent with previous results, methyltransferases and demethylases were detected mostly in the nucleus under normal conditions (Fig. 1B to F). However, METTL3, METTL14, WTAP, ALKBH5, and FTO were all present in both the nucleus and cytoplasm after infection (Fig. 1B to F). The colocalization of methyltransferases and demethylases with viral protein N implied that these proteins may interact with SARS-CoV-2 RNA in the cytoplasm. The above-described results provided evidence that SARS-CoV-2 may be modified by the host m^6^A machinery.

### SARS-CoV-2 RNA contained m^6^A modifications.

**FIG 2:**
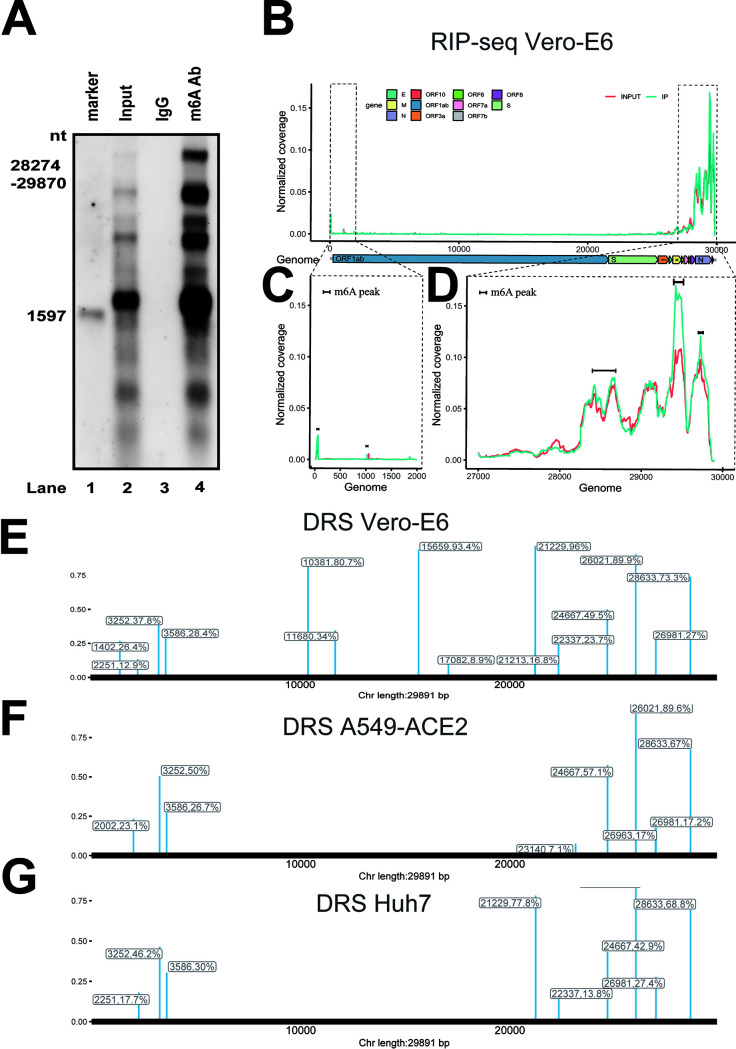
SARS-CoV-2 genomic RNA harbored m^6^A modifications. (A) MeRIP and Northern blotting. RNAs from virus-infected Vero E6 cells were incubated with IgG or anti-m^6^A-specific antibodies as indicated. Immunoprecipitated RNAs were resolved on 1% agarose gels containing 2.2 M formaldehyde and transferred to Hybond-N^+^ membranes, followed by RNA signal detection with SARS-CoV-2-specific probes spanning from nt 28274 to nt 29870. (B to D) MeRIP-Seq. Fragmented total RNAs from SARS-CoV-2-infected Vero E6 cells were subjected to IP with anti-m^6^A-specific antibodies, followed by next-generation sequencing. Methylation coverage of the full-length SARS-CoV-2 RNA is shown. Representative of *n* = 2 determinations. (E to G) DRS (direct RNA sequencing). PolyA-purified mRNAs from SARS-CoV-2-infected Vero E6, A549-ACE2, and Huh7 cells were used to nanopore-direct RNA sequencing and bioinformatic analysis.

To investigate whether SARS-CoV-2 RNA was m^6^A modified, total RNAs were purified from large-scale batches of SARS-CoV-2-infected Vero E6 cells, and MeRIP was then performed with m^6^A-specific antibodies. The MeRIP RNA was subjected to Northern blotting with SARS-CoV-2 probes spanning nucleotides (nt) 28274 to 29870. SARS-CoV-2 RNA was then pulled down using anti-m^6^A antibodies (Fig. 2A), indicating that SARS-CoV-2 contained m^6^A residues. To further confirm the above-described results and map the m^6^A modification status in the SARS-CoV-2 RNA genome, MeRIP-Seq was performed. Five m^6^A peaks were identified in the 5′ end (nt 36 to 753 and nt 1023 to 1324) and the 3′ end (nt 27493 to 27913, nt 28475 to 28706, and nt 28944 to 29751) (Fig. 2B to D), which were located in the ORF1ab-, N-, and ORF10-coding regions (Fig. S1A). These results implied that SARS-CoV-2 RNA was marked by m^6^A modification during infection. To further confirm the specific m^6^A modification sites, we performed the nanopore-based direct RNA sequencing (DRS) using a MinION nanopore sequencer with total RNAs extracted from Vero E6, A549-ACE2, and Huh7 cells infected with SARS-CoV-2. Consistent with the MeRIP-Seq results, most m^6^A sites were distributed in the 5′ and 3′ ends (Fig. 2E to G) in different infected cell lines. Notably, six m^6^A sites were conserved in all of the infected cell lines (Fig. S1B), and the m^6^A motif in the SARS-CoV-2 genome is mainly GGACA (Fig. S1E). Nine m^6^A sites mapped in A549-ACE2 cells were completely included in Vero E6 cells (Fig. S1C). Three m^6^A sites in Huh7 cells different from those in Vero E6 and A549-ACE2 cells (Fig. S1D). These results indicated that both the conserved and different m^6^A sites existed in different cell lines.

10.1128/mBio.01067-21.1 FIG S1 m^6^A modification status of SARS-CoV-2. (A) Table of m^6^A peak regions by MeRIP-seq. The five peak regions are shown. (B) m^6^A sites were mapped by DRS of the SARS-CoV-2 genome in Vero E6, A549-ACE2, and Huh7 cell lines. Six m^6^A sites are conserved. (C) Nine m^6^A sites mapped by DRS in A549-ACE2 cells are completely contained in Vero E6 cells. (D) Three m^6^A sites were mapped by DRS in Huh7 different from those in Vero E6 and A549-ACE2 cells. (E) Analysis of m^6^A motif. The m^6^A motif detected by MINES Motif analysis with DRS data. The horizontal axis is the number of bases at the methylation site, the total height at each position is the sequence conservatism of the base at that position, and the height of the base signal represents the relative frequency of the base at that position. Download FIG S1, TIF file, 1.7 MB. Copyright © 2021 Zhang et al. 2021 Zhang et al. https://creativecommons.org/licenses/by/4.0/ This content is distributed under the terms of the Creative Commons Attribution 4.0 International license.

### METTL3 promoted m^6^A modification of SARS-CoV-2 RNA and virus replication.

**FIG 3:**
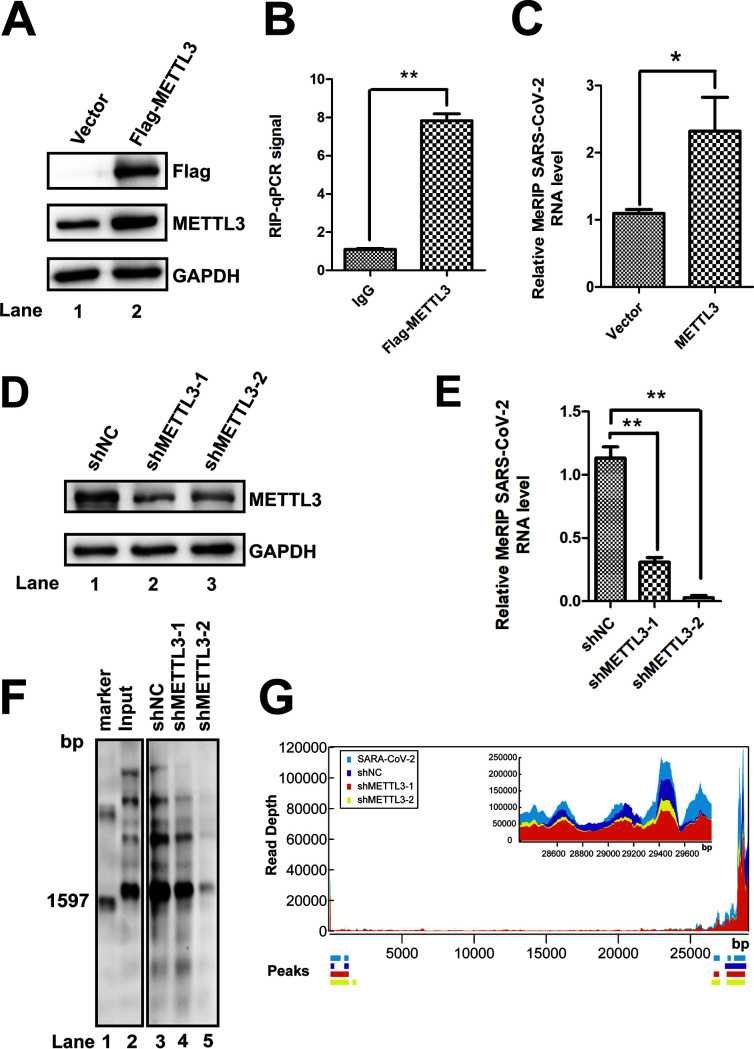
METTL3 catalyzed the m^6^A modification of SARS-CoV-2. (A and D) Western blotting. *METTL3* was knocked down by shRNA (D) or overexpressed (A) in Vero E6 cells. The expression of METTL3 was checked using anti-METTL3 (A and D) or anti-Flag antibodies (A) as indicated. Vector-transfected cells were used as a control. (B) Formaldehyde-RIP qRT-PCR. Cell lysates from formaldehyde-cross-linking were subjected to IP with IgG or anti-Flag antibodies. qRT-PCR was performed to quantify SARS-CoV-2 RNA. IgG was used as a negative control. Unpaired Student’s *t* tests were performed, and the data are presented as means ± standard errors of the means (*n* = 3). **, *P ≤ *0.01. (C and E) MeRIP-qPCR. RNA was extracted from SARS-CoV-2-infected Vero E6 cells in which *METTL3* was overexpressed (C) or knocked down by shRNA (E). MeRIP was performed, and SARS-CoV-2 RNA was quantified by qRT-PCR. Unpaired Student’s *t* tests were performed, and data are presented as means ± standard errors of the means (*n* = 3). **, *P ≤ *0.01. (F) MeRIP and Northern blotting. RNAs were harvested from SARS-CoV-2-infected Vero E6 cells in which *METTL3* was knocked down by shRNA. (G) MeRIP-Seq. Total RNA was isolated from SARS-CoV-2-infected Vero E6 cells in which METTL3 was knocked down.

The host methyltransferases and demethylases are involved in the m^6^A modification of EV71, HCV, ZIKV, and HIV because these viruses do not encode any enzymes with m^6^A methyltransferase activity (37–41, 50, 53). To determine whether host m^6^A machinery was responsible for the SARS-CoV-2 m^6^A modification, the FLAG-tagged *METTL3* gene was expressed in Vero E6 cells by transfection (Fig. 3A). Quantitative reverse transcription PCR (qRT-PCR) of the *RdRp* gene was performed following formaldehyde-cross-linked RNA immunoprecipitation (RIP) using an anti-FLAG antibody to pull down METTL3-bound RNAs. SARS-CoV-2 RNAs were pulled down by METTL3 (Fig. 3B), indicating that SARS-CoV-2 RNA could interact with METTL3. We next knocked down endogenous *METTL3* in Vero E6 cells using short hairpin RNA (shRNA) (Fig. 3D). m^6^A abundance in SARS-CoV-2 RNA was detected by using qRT-PCR (Fig. 3E) or Northern blotting (Fig. 3F) after MeRIP. We found that silencing METTL3 by shRNA resulted in decreased abundance of m^6^A in SARS-CoV-2 RNA (Fig. 3D and E). In contrast, overexpression of METTL3 by transfection increased the abundance of m^6^A-bound SAR-CoV-2 RNAs (Fig. 3C). To further confirm our results, MeRIP-Seq was performed after *METTL3* knockdown (Fig. 3G and Fig. S6). Our results showed that the methylation peaks were not changed, but that the frequency of methylation was significantly decreased, suggesting that the m^6^A modification levels in the SARS-CoV-2 genome were linked to METTL3 expression. Taken together, these results indicated that METTL3 acted as a methyltransferase in the viral genome.

10.1128/mBio.01067-21.6 FIG S6 Effect of METTL3 gene knockdown in m^6^A methylation levels. Total RNAs were isolated from SARS-CoV-2-infected Vero E6 cells in which METTL3 was knocked down and then subjected to IP with m^6^A-specific antibody, followed by next-generation sequencing. In order to make the samples comparable, IP reads mapping to the virus genome were normalized to the total number of sequenced reads. Peaks represent m^6^A-enriched regions detected by MACS2. Download FIG S6, TIF file, 0.7 MB. Copyright © 2021 Zhang et al. 2021 Zhang et al. https://creativecommons.org/licenses/by/4.0/ This content is distributed under the terms of the Creative Commons Attribution 4.0 International license.

**FIG 4:**
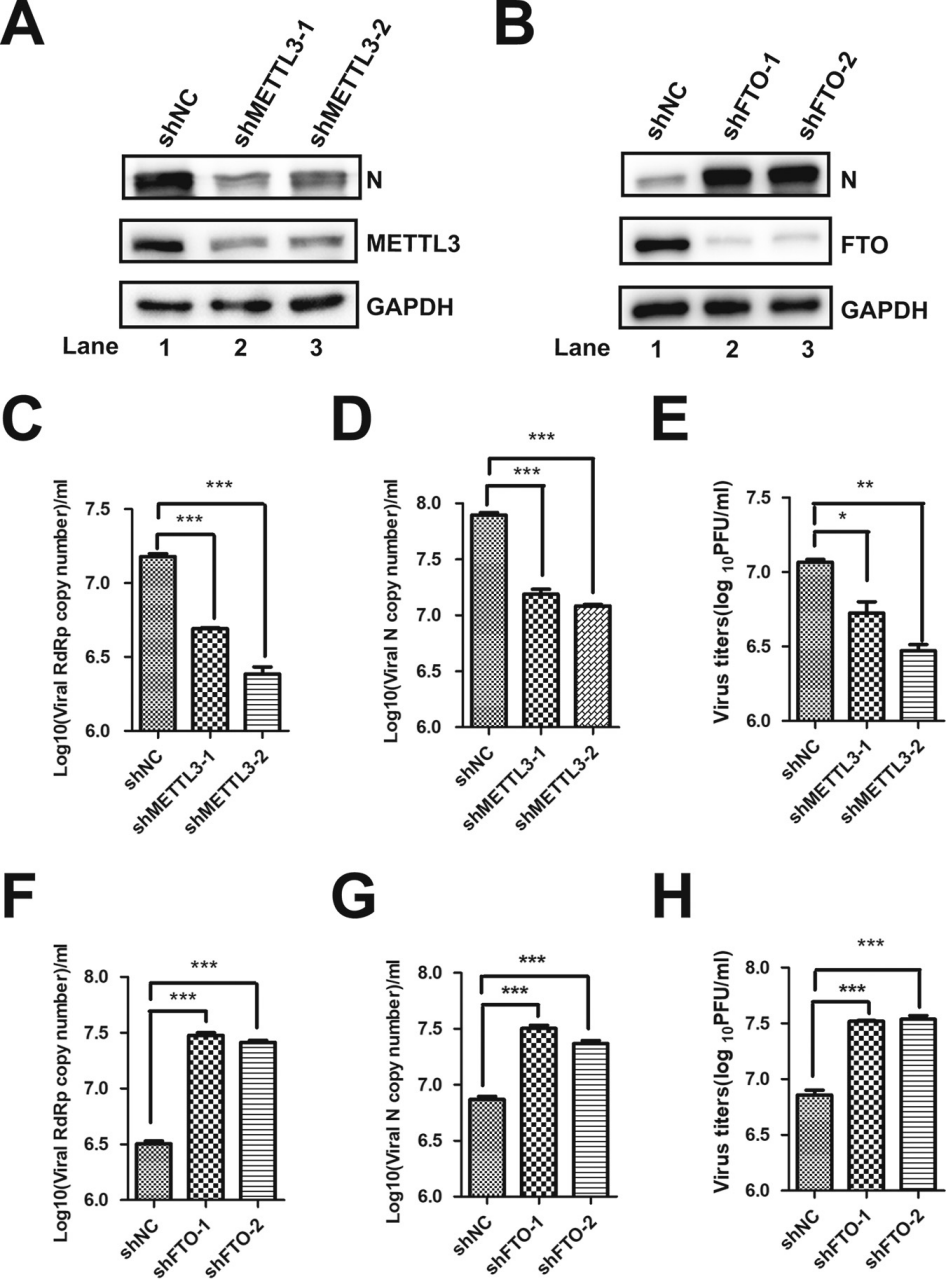
METTL3- and FTO-regulated SARS-CoV-2 replication. (A and B) Western blotting. *METTL3* and *FTO* were knocked down in Vero E6 cells by shRNA. The expression of METTL3, FTO, and viral N protein were detected by Western blotting with specific antibodies. (C and D, F, and G) qRT-PCR. Total RNA was isolated from SARS-CoV-2-infected Vero E6 cells in which *METTL3* and *FTO* was knocked down by shRNA as indicated. SARS-CoV-2 RNA was quantified using qRT-PCR with specific primers targeting *N* and *RdRp* genes. *GAPDH* was used as a control. Unpaired Student’s *t* tests were performed. Data are presented as means ± SEMs (*n* = 3). *, *P ≤ *0.05. (E and H) Viral titers. Vero E6 cells in which *METTL3* and *FTO* were knocked down were infected by SARS-CoV-2, and the supernatants were collected at 24 h postinfection to measure virus titers by plaque assay.

Viral protein expression and progeny virus production by HIV, HCV, and EV71 are affected by the expression of endogenous methyltransferases or demethylases (37, 40, 50). Endogenous *METTL3* (Fig. 4A) or FTO (Fig. 4B) in Vero E6 cells was knocked down by specific shRNAs, followed by SARS-CoV-2 infection to check whether METTL3 or FTO affected viral replication. Viral titer was measured by plaque assay, and RNA copy numbers were quantified by qRT-PCR of the *N* or *RdRp* gene using standard protocols. We found that efficient knockdown of *METTL3* not only resulted in significant decreases in virus titer (Fig. 4E) and viral *N* and *RdRp* gene copy numbers (Fig. 4C and D) but also decreased expression of N (Fig. 4A). However, knockdown of *FTO* had the opposite effect (Fig. 4B and F to H). These results suggested that the m^6^A methyltransferase METTL3 was linked to efficient SARS-CoV-2 replication.

### SRAS-CoV-2 RNA-dependent RNA polymerase (RdRp) interacted with METTL3 and facilitated its expression.

**FIG 5:**
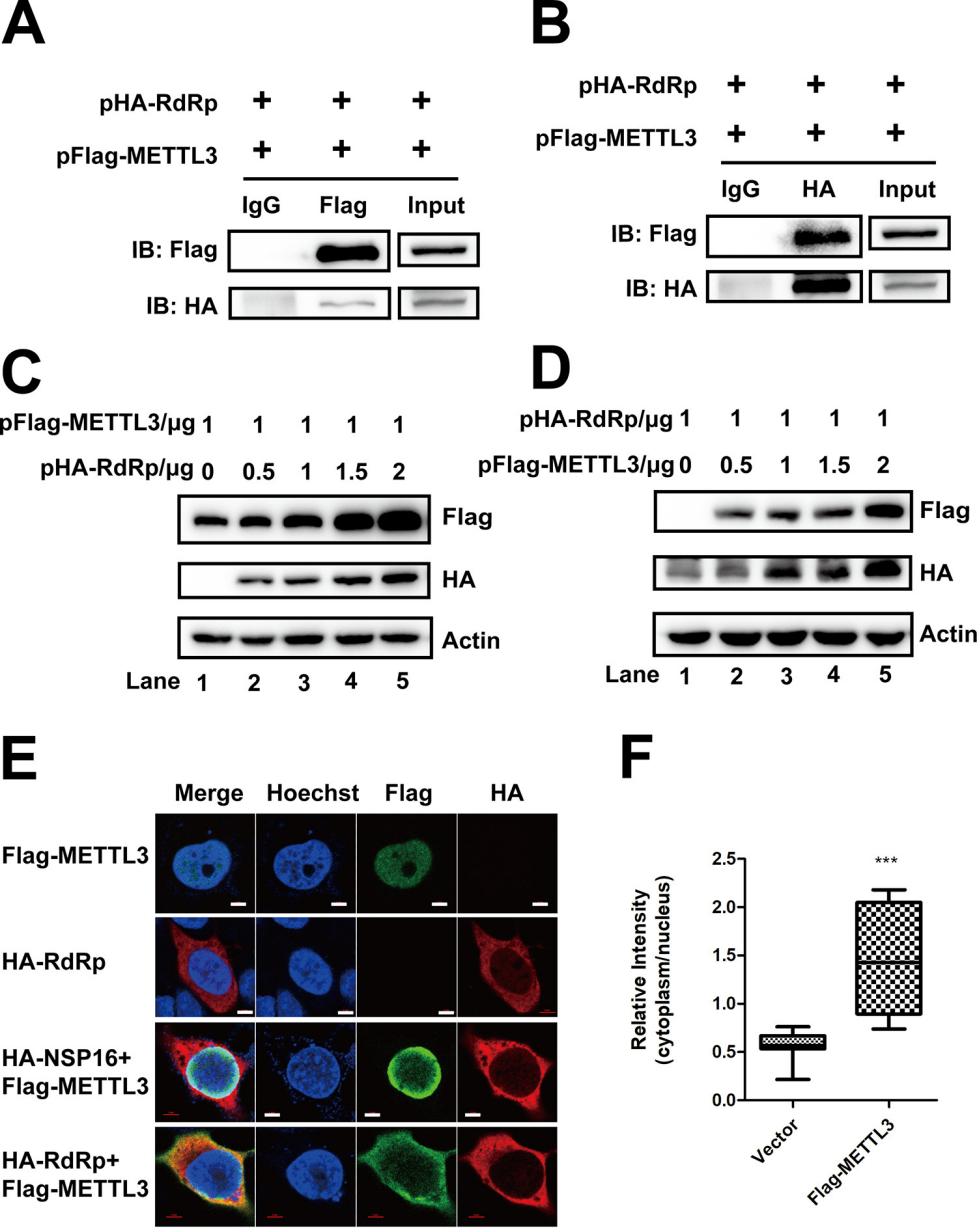
SRAS-CoV-2 RdRp interacted with METTL3 and influenced its expression. (A and B) Western blotting. pFlag-METTL3 and pHA-RdRp were cotransfected into Huh7 cells, and co-IP was performed with anti-HA (A) or anti-Flag (B) antibodies. IgG antibodies were used as a control. The IP samples were pulled down with anti-Flag (A) or anti-METTL3 (B) antibodies. (C and D) Western blotting. Huh7 cells were transfected with 1 μg pFlag-METTL3 (C) or pHA-RdRp (D) together with different amounts of pHA-RdRp (C) or pFlag-METTL3 (D) (0, 0.5, 1, 1.5, and 2 μg, respectively) in six-well plates. The expression of METTL3 and RdRp was detected by Western blotting. (E) Confocal microscopy images. Huh7 cells were transfected with pFlag-METTL3 with or without HA-RdRp and HA-NSP16 transfection. Costaining was performed using anti-Flag (green) and anti-HA antibodies (red). The nucleus (blue) was stained with Hoechst. (F) Relative fluorescence intensity of METTL3 in the cytoplasm versus the nucleus was quantified using ImageJ and graphed in box-and-whisker plots, representing the minimum, first quartile, median, third quartile, and maximum. Unpaired Student’s *t* test was performed (*n* ≥ 10). ***, *P* ≤ 0.001.

**FIG 6:**
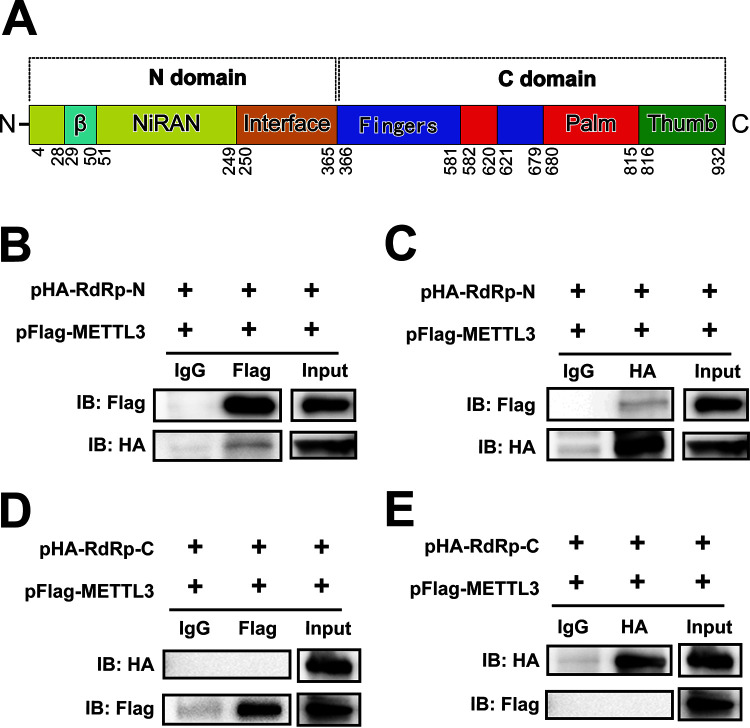
The N terminus of RdRp interacted with the METTL3. (A) Schematic diagram of the RdRp domain. The N-terminal and C-terminal domains consist of 1-365AA and 366-932AA, respectively. (B to E) Western blotting. Huh7 cells were cotransfected with pFlag-METTL3 and pHA-RdRp-N (B and C) or pHA-RdRp-C (D and E). Co-IP was performed with anti-HA (C and E) or anti-Flag (B and D) antibodies. IgG antibody was used as a control. The immunoblots were visualized by the indicated antibodies.

In our previous study, METTL3 modulated EV71 replication by interacting with EV71 polymerase 3D and regulating 3D sumoylation and ubiquitination (50). To investigate whether there was a similar mechanism in SARS-CoV-2, pFlag-METTL3 and pHA-RdRp were cotransfected into Huh7 and HEK293T cells. The IP experiment with anti-Flag antibodies, followed by staining with anti-HA or vice versa, showed that METTL3 interacted with SARS-CoV-2 RdRp protein in the absence or presence of RNase A (Fig. 5A and B, Fig. S2A and B). In addition, our study showed that RdRp interacted with the methyltransferase complex (Fig. S4A to D). To determine the functional domain interacting with METTL3, the N and C termini of RdRp were cloned and cotransfected with pFlag-METTL3 into Huh7 cells (Fig. 6A). We found that METTL3 interacted with RdRp-N (Fig. 6B and C) but not RdRp-C (Fig. 6D and E). To further confirm our results, the colocalization of METTL3 and RdRp was checked after cotransfection of the cells with the two plasmids. Notably, METTL3 was distributed in both the nucleus and cytoplasm when RdRp was coexpressed (Fig. 5E and F). However, the coexpression of nonstructural protein NSP16 of SARS-CoV-2 had no effect on the subcellular localization of METTL3. The colocalization of METTL3 and RdRp supported the interaction between these two proteins, which bound to viral RNAs. Moreover, METTL3 expression was increased as more RdRp was expressed by transfection or vice versa (Fig. 5C and D), suggesting that the abundance of METTL3 and RdRp influenced the expression of RdRp or METTL3.

10.1128/mBio.01067-21.2 FIG S2 METTL3 interacted with SARS-CoV-2 RdRp in an RNA-independent way. (A and B) Western blotting. pFlag-METTL3 and pHA-RdRp were cotransfected into HEK293T cells, and cell extracts were digested with RNaseA for 15 min at 37°C. Co-IP was performed with anti-Flag (A) or anti-HA (B) antibodies. IgG antibody was used as a control. The immunoblots were visualized by the indicated antibodies. Download FIG S2, TIF file, 0.5 MB. Copyright © 2021 Zhang et al. 2021 Zhang et al. https://creativecommons.org/licenses/by/4.0/ This content is distributed under the terms of the Creative Commons Attribution 4.0 International license.

10.1128/mBio.01067-21.4 FIG S4 SARS-CoV-2 RdRp interacted with the methyltransferase complex. (A to D) Western blotting. HEK293T cells were cotransfected with pMETTL14 and pHA-RdRp (A and B) or pFlag-WTAP and pHA-RdRp (C and D).Co-IP was performed with anti-METTL14 (A) or anti-HA (B and C) or anti-Flag (D) antibodies. IgG antibody was used as a control. The immunoblots were visualized by the indicated antibodies. Download FIG S4, TIF file, 1 MB. Copyright © 2021 Zhang et al. 2021 Zhang et al. https://creativecommons.org/licenses/by/4.0/ This content is distributed under the terms of the Creative Commons Attribution 4.0 International license.

### SRAS-CoV-2 RdRp inhibited METTL3 sumoylation and ubiquitination.

**FIG 7:**
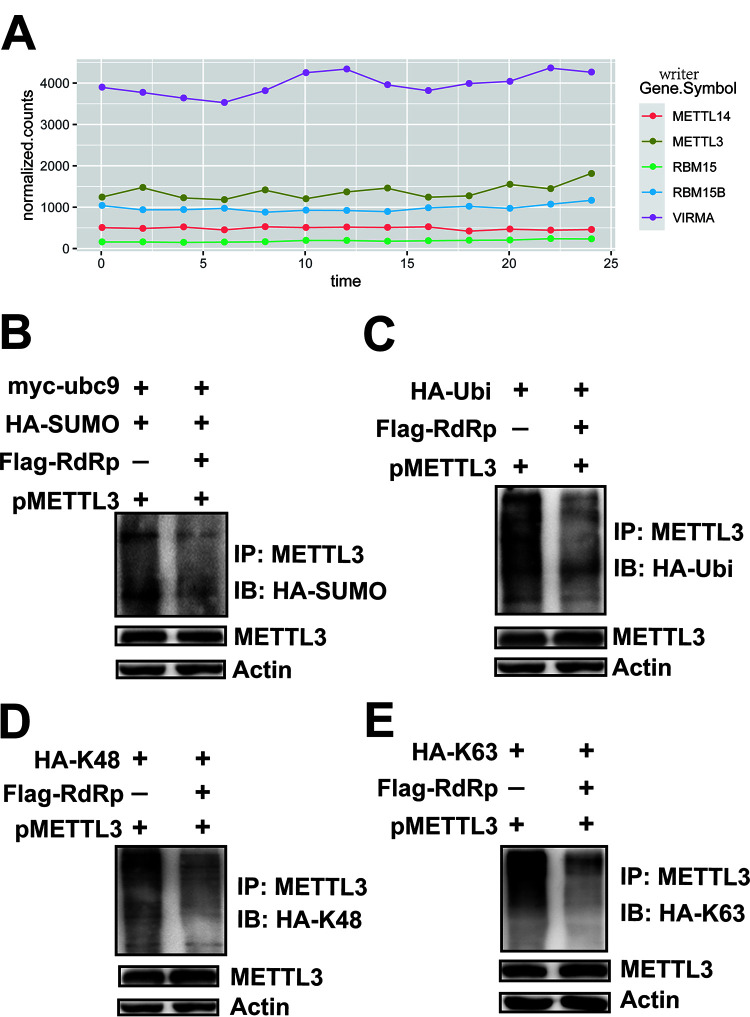
RdRp expression inhibited the sumoylation and ubiquitination of METTL3. (A) RNA expression of host methyltransferases. Total RNA was harvested from SARS-CoV-2-infected Vero E6 cells every 2 h as indicated. The mRNAs were separated and subjected to next-generation sequencing. RNA levels of host methyltransferases were normalized according to the sequencing reads. (B) Sumoylation assay. *METTL3* was overexpressed in HEK293T cells by transfection with pMETTL3, followed by transfection with pFlag-RdRp, pHA-SUMO-1, and pMyc-Ubc9. IP and immunoblot analyses were performed using the indicated antibodies for the sumoylation assay. (C to E) Ubiquitination assay. HEK293T cells were transfected with pFlag-RdRp, pHA-Ubi, pHA-K48, and pHA-K63 after *METTL3* overexpression. IP and immunoblot analyses were performed using the indicated antibodies.

To analyze how viral protein RdRp affected the m^6^A machinery components, we first checked the RNA abundance of all the m^6^A writers, erasers, and readers after different infection times as indicated (Fig. 7A and Fig. S5). The RNA abundances of *METTL3*, *METT14*, *WTAP*, *ALKBH5*, *FTO*, and *YTH* were not changed (Fig. 7A), indicating that SARS-CoV-2 did not influence the RNA levels of m^6^A-related proteins. Posttranscriptional modifications, such as ubiquitination and sumoylation, affect METTL3 protein abundance and function. EV71 3D protein interacted with METTL3 and affected the expression and localization METTL3, similar to the results observed for SARS-CoV-2 infection. We next investigated whether RdRp affected the modification of METTL3. To this end, METTL3, pFlag-RdRp, HA-SUMO-1, and myc-Ubc-9 were transfected into HEK293T cells. The Western blotting results showed that sumoylation of METTL3 was reduced in the presence of RdRp expression (Fig. 7B and Fig. S3A). Cotransfection with pMETTL3, Flag-RdRp, and HA-Ub resulted in decreased ubiquitination of METTL3 (Fig. 7C and Fig. S3B). Further experiments showed that overexpression of RdRp resulted in decreased K48-linked ubiquitination (Fig. 7D and Fig. S3C) and K63-linked ubiquitination (Fig. 7E and Fig. S3D). However, the viral nonstructural protein NSP16 overexpression had no effect on posttranslational modifications of the METTL3 (Fig. S3A to D).

10.1128/mBio.01067-21.3 FIG S3 RdRp expression inhibited the sumoylation and ubiquitination of METTL3 in Huh7 cells. (A) Sumoylation assay. METTL3 was overexpressed in Huh7 cells by transfection with pMETTL3, followed by transfection with pHA-SUMO-1, pmyc-Ubc9, and pFlag-RdRp or pFlag-NSP16. IP and immunoblot analyses were performed using the indicated antibodies for the sumoylation assay. (B to D) Ubiquitination assay. Huh7 cells were transfected with pHA-Ubi, pHA-K48, pHA-K63, and pFlag-RdRp or pFlag-NSP16 after METTL3 overexpression. IP and immunoblot analyses were performed using the indicated antibodies. Download FIG S3, TIF file, 1.8 MB. Copyright © 2021 Zhang et al. 2021 Zhang et al. https://creativecommons.org/licenses/by/4.0/ This content is distributed under the terms of the Creative Commons Attribution 4.0 International license.

10.1128/mBio.01067-21.5 FIG S5 RNA expression of host demethylases and m^6^A binding proteins. Total RNA was harvested from SARS-CoV-2-infected Vero E6 cells every 2 h as indicated. The mRNAs were separated and subjected to next-generation sequencing. The RNA level of host demethylases and m^6^A binding proteins were normalized according to the counts. Download FIG S5, TIF file, 0.8 MB. Copyright © 2021 Zhang et al. 2021 Zhang et al. https://creativecommons.org/licenses/by/4.0/ This content is distributed under the terms of the Creative Commons Attribution 4.0 International license.

## DISCUSSION

In the current study, we demonstrated that SARS-CoV-2 RNA underwent m^6^A modification by host m^6^A machinery. The expression and localization of host m^6^A components were altered during SARS-CoV-2 infection. Knockdown of METTL3 decreased the replication of SARS-CoV-2, indicating that m^6^A modification played key roles in viral replication. Further studies showed that the viral polymerase RdRp interacted with METTL3 and regulated its sumoylation and ubiquitination to affect its expression and localization. Overall, our study showed that SARS-CoV-2 RNA was m^6^A modified and that METTL3 played a role in regulating viral replication.

RNA modification, such as m^6^A, m^5^C, and ac4C, regulates viral protein expression and progeny virus production (5–10). m^6^A modification has been identified in RNA viruses replicating in both the nucleus and the cytoplasm (5, 9, 54) and has different regulation mechanisms. Similar to the RNA viruses influenza A virus (IAV), human immunodeficiency virus (HIV), and enterovirus 71 (EV71) (40, 42, 50, 51), the m^6^A modification of SARS-CoV-2 promotes virus replication in Vero E6 cells, which is different from the result of SARS-CoV-2 infection in Huh7 cells (47). The m^6^A modification of viral RNAs attenuates host innate immunity via RIG-I signaling in virus infection, indicating that the interferon pathway is linked to the viral m^6^A modification (55–57). As Vero E6 cells are immunodeficient, the regulatory mechanism in our research may be different from the reported literature.

The location and pattern of m^6^A between virus and host RNA are different. In our study, we assessed the internal m^6^A modification status of SARS-CoV-2 RNA by MeRIP-Seq and DRS. The results demonstrated that m^6^A peaks were mainly distributed in both 5′ and 3′ ends spanning the ORF1ab-, N-, and ORF10-encoding regions in different cell lines. The m^6^A modification pattern of SARS-CoV-2 is very similar to that of host mRNAs (12, 58) but different from that of EV71, whose m^6^A sites are distributed in coding regions of the middle of the viral genome (50). Notably, 44 m^6^A motifs were found in the 5 enriched peaks, most of which were distributed in the N gene region.

SARS-CoV-2 infection resulted in not only elevated expression of METTL3 but also altered distribution in both the nucleus and cytoplasm. We also found that METTL14, WTAP, ALKBH5, and FTO colocalized with the viral protein N, supporting the finding that SARS-CoV-2 infection affected the m^6^A methyltransferase and demethylases. The colocalization of viral N and host m^6^A proteins supported the finding that the m^6^A modification machinery could modify cytoplasmic SARS-CoV-2 RNA during infection. In our previous study, we found that METTL3 interacted with EV71 polymerase 3D protein (50). Cotransfection of cells with METTL3 and 3D resulted in both nuclear and cytoplasmic distribution of METTL3, implying that 3D played roles in the distribution of METTL3 in the cytoplasm; however, the specific mechanism is still unknown. In the current study, we found that the SARS-CoV-2 RdRp protein induced the expression and cytoplasmic distribution of METTL3.

Most RNA viruses that replicate in the cytoplasm, including ZIKV, West Nile virus, PEDV, and EV71, hijack the host m^6^A machinery to modify the RNA and therefore do not encode methyltransferase (37, 38, 50, 52). SARS-CoV-2 nonstructural proteins NSP14 and NSP16 have methyltransferase function and play key roles in the m^7^G cap and 2′-*O*-methylation modification (59–61). Our study showed that METTL3 interacted with SARS-CoV-2 RNA. Notably, the expression of METTL3 is linked to the m^6^A modification level of SARS-CoV-2 RNA. Knocking down METTL3 resulted in decreased m^6^A modification of SARS-CoV-2 RNA, which was detected either by MeRIP Northern blotting or by MERIP-Seq. However, overexpression of METTL3 resulted in elevated m^6^A modification, suggesting that METTL3 may be the methyltransferase modifying viral RNA. METTL3 is a multifunctional protein that functions during EV71 infection. Viral RdRp 3D protein binds to the methyltransferase complex, and METTL3 regulates the ubiquitination of 3D to promote viral replication (50). In this study, knockdown of METTL3 resulted in decreased SARS-CoV-2 replication; this result could be explained by the absence of METTL3 methyltransferase activity, which catalyzes the methylation of viral RNA. To address whether key proteins of SARS-CoV-2 interacted with m^6^A components to facilitate virus replication, we checked the interactions of METTL3 with viral RdRp, which bound to viral RNA. The results showed that METTL3 not only interacted with RdRp but also promoted RdRp expression; the opposite result was also true. In contrast to EV71 3D protein, for which posttranslational modification was modulated by METTL3, SARS-CoV-2 RdRp expression altered the localization pattern of METTL3. The distribution of METTL3 in the presence of RdRp expression confirmed the interaction between METTL3 and RdRp and may explain the presence of METTL3 in the cytoplasm during SARS-CoV-2 infection. Sumoylation and ubiquitination affect the function and expression of METTL3, respectively (62). To elucidate how RdRp expression increased the expression of METTL3, we checked the protein modification of METTL3. The results showed that RdRp expression decreased the sumoylation and overall ubiquitination levels. Moreover, K48- and K63-linked ubiquitination levels were reduced. These data supported that RdRp not only promoted methyltransferase activity but also increased METTL3 expression by decreasing its ubiquitination.

In summary, our results provided evidence that the host m^6^A machinery interacted with viral key proteins to facilitate the replication of SARS-CoV-2. First, METTL3 functioned as a methyltransferase, adding the m^6^A modification to viral RNA. Second, METTL3 interacted with viral RdRp, which resulted in METTL3 distribution both in the nucleus and in the cytoplasm. Importantly, RdRp boosted the expression of METTL3 by altering the ubiquitination pattern through an unknown mechanism. Further studies are required to elucidate this mechanism. The functional m^6^A sites on the SARS-CoV-2 RNA need to be defined on the infectious clone to further verify the influence of m^6^A modification on virus replication.

### Data availability.

SARS-CoV-2 sequence data that support the findings of this study have been deposited in GISAID (https://www.gisaid.org/) with the accession numbers EPI_ISL_402124, EPI_ISL_402127 to EPI_ISL_402130, and EPI_ISL_402131; in GenBank with accession numbers MN996527 to MN996532; and in the National Genomics Data Center, Beijing Institute of Genomics, Chinese Academy of Sciences (https://bigd.big.ac.cn/databases?lang=en) with accession numbers SAMC133236 to SAMC133240 and SAMC133252.

## MATERIALS AND METHODS

### Virus, cell lines, and cell culture.

SARS-CoV-2 (IVCAS 6.7512) was obtained from the Virus Resource Center of the Wuhan Institute of Virology of the Chinese Academy of Sciences and passaged in monkey kidney cells (Vero E6 cells) for eight generations. The titer of the SARS-CoV-2 working solution was 10^6^ PFU/ml, as determined by plaque assays in Vero E6 cells. Vero E6 cells (American Tissue Culture Collection [ATCC], Manassas, VA, USA; CRL-1586), and HEK293T cells (ATCC; CRL-11268), A549-ACE2 (ATCC, CCL-185), and Huh7 liver hepatocellular cancer cells (obtained from the Wuhan Institute of Virology of the Chinese Academy of Sciences) were cultured in Dulbecco’s modified Eagle’s medium 116 (Gibco, Gaithersburg, MD, USA) containing 10% fetal bovine serum (Gibco) at 37°C with 5% CO2.

### Plasmid construction and transfection.

The RNA-dependent RNA polymerase (RdRp) plasmids and nonstructural protein 16 (NSP16) pFlag-RdRp, pHA-RdRp, pFlag-NSP16, and pHA-NSP16 were constructed by inserting the sequences of the RdRp and NSP16 open reading frame (ORF) into the vectors pXJ40-Flag and pXJ40-HA (Sigma-Aldrich, St. Louis, MO, USA), respectively. The N- and C-terminal domains of RdRp were also cloned into the vector pXJ40-HA. m^6^A methyltransferases and demethylase expression plasmids (pFlag-METTL3, pFlag-WTAP, pMETTL3, pMETTL14, and Flag-METTL3) were constructed by inserting the ORF sequences of the genes into the vector pXJ40-Flag, pcDNA3.0, or pLenti-CMV-3XFlag. The plasmids HA-SUMO-1, HA-Ubi, HA-63, HA-48, and myc-Ubc9 were kind gifts from Hanzhong Wang (Wuhan Institute of Virology, Chinese Academy of Sciences [CAS]).

Plasmids were transfected into cells using Lipofectamine 2000 reagent (Invitrogen, Carlsbad, CA, USA; catalog [cat.] no. 11668-019) according to the manufacturer’s instructions.

### Western blotting and antibodies.

Cell lysates were prepared at the indicated times after transfection or infection and separated by gradient sodium dodecyl sulfate polyacrylamide gel electrophoresis (SDS-PAGE) on 10% gels, and proteins were then transferred to nitrocellulose membranes. The membranes were incubated with primary antibodies overnight at 4°C at the dilution suggested by the manufacturer’s protocols. The primary antibodies were as follows: anti-glyceraldehyde 3-phosphate dehydrogenase (GAPDH; cat. no. 60004-1-lg; Proteintech, Rosemont, IL, USA), mouse monoclonal anti-β-actin (cat. no. sc47778; Santa Cruz Biotechnology, Dallas, TX, USA), rabbit monoclonal anti-METTL3 (cat. no. 15073-1-AP; Proteintech), anti-METTL3 (cat. no. ab195352; Abcam, Cambridge, UK), anti-METTL14 (cat. no. SAB1104405; Sigma-Aldrich), anti-WTAP (cat. no. ab155655; Abcam), anti-ALKBH5 (cat. no. ab69325; Abcam), anti-FTO (cat. no. ab124892; Abcam), anti-Flag (cat. no. F1804-1 MG; Sigma-Aldrich), anti-HA (cat. no. H9658; Sigma-Aldrich), and mouse polyclonal anti-SARS-CoV-2 nonstructural protein (NP) (gift from Fei Deng, Wuhan Institute of Virology, CAS). The secondary antibodies, including goat anti-mouse IgG and goat anti-rabbit IgG (AntiGene Biotech GmbH, Stuttgart, Germany) were incubated for 1 h at a dilution of 1:5,000. Luminescent signals were detected using a Tanon-5200 ChemiDoc MP imaging system (Tanon Science & Technology, Shanghai, China).

### Coimmunoprecipitation.

Total proteins were collected 48 h after transfection. Primary antibodies were mixed with supernatants of cell lysates (2 μg primary antibody per 1 mg protein sample) for 2 h at 4°C and then incubated with protein G agarose overnight at 4°C. Immunoprecipitated proteins were separated by SDS-PAGE on 12% gels and transferred nitrocellulose membranes, followed by incubation with primary and second antibodies. Protein detection was performed using a Tanon 5200 ChemiDoc MP imaging system.

### Short hairpin RNA (shRNA) knockdown.

First, shRNA knockdown was performed according to the protocol for shRNA-mediated gene silencing and lentiviral particle packaging from the Addgene website. Stable-knockdown Vero E6 cell lines were screened using 10 μg/ml puromycin for selection. shRNA-specific primers were as follows: *METTL3* (shMETTL3-1: 5′-GCCAAGGAACAATCCATTGTT-3′, shMETTL3-2: 5′-CGTCAGTATATTGGGCAAGTT-3′), *FTO* (shFTO-1: 5′-TCACCAAGGAGACTGCTATTT-3′, shFTO-2: 5′-GATCCAAGGCAAAGATTTACT-3′).

### Immunofluorescence analysis.

Immunofluorescence analyses were performed as previously described (63). Briefly, Vero E6 cells were infected with SARS-CoV-2 (multiplicity of infection [MOI] = 0.01) and harvested 24 h postinfection. Cells were fixed in 4% paraformaldehyde overnight, permeabilized in 0.2% Triton X-100 for 10 min, washed three times with phosphate-buffered saline (PBS), and blocked in 3% bovine serum albumin for 1 h at room temperature. The cells were incubated with primary antibodies overnight at 4°C at the dilution suggested by the manufacturer’s protocol and stained with secondary antibodies (Alexa Fluor 488, Alexa Fluor 568) for 1 h after three washes with PBS. Nuclei were visualized with Hoechst 33258 at a dilution of 1:1,000. The images were captured under a PerkinElmer VoX confocal microscope.

### Quantitative reverse transcription PCR (qRT-PCR).

Total RNA was extracted from SARS-CoV-2-infected Vero E6 cells, and reverse transcription was performed using a HiScript first-strand cDNA synthesis kit (Vazyme Biotech Co., Nanjing, China) according to the manufacturer’s instructions, followed by quantitative PCR with SYBR green (Yeasen Biotech Co., Shanghai, China) on a CFX Connect real-time system (Bio-Rad Laboratories, Hercules, CA, USA). *GAPDH*, *N*, and *RdRp* gene-specific primers are described in Table S1.

10.1128/mBio.01067-21.7 TABLE S1 Information of *GAPDH*, *N*, and *RdRp* gene-specific primers. Download Table S1, DOCX file, 0.01 MB. Copyright © 2021 Zhang et al. 2021 Zhang et al. https://creativecommons.org/licenses/by/4.0/ This content is distributed under the terms of the Creative Commons Attribution 4.0 International license.

### Plaque assay.

SARS-CoV-2 was propagated on the Vero E6 cells and titrated by single-layer plaque assay with a standard procedure. Briefly, Vero E6 cells were seeded into 24-well plates at a concentration of 1 × 10^5^ cells per well. Then, 24 h later, confluent Vero E6 cells were infected with 200 μl of DMEM containing a serial 10-fold dilution of viral stock for 1 h at 37°C. After removal of the inoculum, Vero E6 cells were overlaid with DMEM medium containing 0.9% methylcellulose and cultured at 37°C for 4 days. Plaques were monitored and counted.

### Formaldehyde-cross-linked RNA-immunoprecipitation (RIP).

RIP was conducted as previously described (50). Briefly, infected Vero E6 cells were cross-linked by adding PBS containing 1% methanol-free formaldehyde and incubated for 10 min at 37°C. The reaction was terminated by adding 2.5 M glycine, and the cells were lysed with 400 μl RIP buffer (150 mM KCl, 25 mM Tris-HCl [pH 7.4], 5 mM ethylenediaminetetraacetic acid [EDTA], 0.5 mM dithiothreitol [DTT], 0.5% NP-40, 100 U/ml RNase inhibitor, 100 μM phenylmethylsulfonyl fluoride [PMSF], and 1 μg/ml proteinase inhibitor) on ice for 10 min. The lysates were then centrifuged at 16,000 × *g* for 10 min, and supernatants were subjected to IP with IgG or anti-Flag antibodies overnight. Next, 30 μl protein-G agarose beads was added after washing three times with washing buffer (300 mM KCl, 25 mM Tris-HCl [pH 7.4], 5 mM EDTA, 0.5 mM DTT, 0.5% NP-40, 100 U/ml RNase inhibitor, 100 μM PMSF, and 1 μg/ml proteinase inhibitor) and incubated with the indicated antibodies for 2 h at 4°C. RNA isolation was performed using TRIzol (Invitrogen, Carlsbad, CA, USA) for qRT-PCR.

### MeRIP-Seq.

Total RNA was extracted from SARS-CoV-2-infected Vero E6 cells and purified with an Oligo (dT) kit (Thermo Scientific, Wilmington, DE, USA). The polyA purified RNA was fragmented and subjected to IP using an m^6^A-specific antibody, followed by next-generation sequencing. MeRIP-Seq data were analyzed as described previously (44). Briefly, reads were quality-checked with FastQC v0.11.8 (http://www.bioinformatics.babraham.ac.uk/projects/fastqc), and then fastp v0.20.1 (64) was used to trim and filter low-quality reads. HISAT 2 (65) was used to align reads to the SARS-CoV-2 reference genome NC_045512. IP and input-normalized coverage were reported in counts per million mapped reads (CPM) using bamCoverage from deepTools (66), with the parameters –binSize 1 –effectiveGenomeSize 29903 –normalizeUsing CPM -p max/2. To make reads depth of IP comparable among the four samples, we normalized IP reads mapping to the virus genome to the total number of sequenced reads to eliminate library differences. IP over input peaks were detected using MACS2 v2.2.7.1 (67), callpeak, using the parameters -f BAMPE -B -g 29903 –nomodel –extsize 200 –scale-to small –bdg –keep-dup=1 -q 0.001.

### Nanopore direct RNA sequencing (DRS).

DRS was described previously (49). First, 1 mg total RNA was extracted from SARS-CoV-2-infected Vero E6, A549-ACE2, and Huh7 cells and purified with an Oligo (dT) kit (Thermo Scientific, Wilmington, DE, USA). Then RNA samples were library prepared following the manufacturer’s instructions (the Oxford Nanopore DRS protocol, SQKRNA002) and loaded on a FLO-MIN106D flow cell, followed by a 48-h sequencing run on a MinION device (Oxford Nanopore Technologies).

We used the sequence of the Wuhan-Hu-1 strain (IVCAS 6.7512) as the viral reference genome, and the nanopore direct sequencing data was analyzed by BENAGEN (Nanopore Company). A threshold with a Q value of 7 was set to obtain pass reads, and base-calling was performed using guppy v3.4.5 (Oxford Nanopore Technologies). The multi_to_single_fast5 of ont_fast5_api (v3.1.6; https://github.com/nanoporetech/ont_fast5_api) was used to convert multi-fast5 reads to single reads, followed by the MINES analysis process (68). First, we used tombo (v1.5; 69) to resquiggle (default parameters) the fast5 data. The *de novo* noncanonical base method mode (tombo detect_modifications de_novo –coverage-dampen-counts 0 0) was applied to detect the bases at each position on the viral genome to check the methylation ratio and coverage of each base. The m6A site was identified using MINES (cDNA_MINES.py, default parameters). Finally, the logo of the m6A motif was drawn with visual analysis using the ggseqlogo package(vggseqlogo_0.1) (70).

### MeRIP and Northern blotting.

For MeRIP and Northern blotting, 400 μg total RNA from virus-infected Vero E6 cells was incubated with an anti-m^6^A antibody (Synaptic Systems, Gottingen, Germany) or an IgG antibody in 300 μl IP buffer (150 mM NaCl, 0.1% NP-40, 10 mM Tris-HCl [pH 7.4]) for 2 h at 4°C. Then 35 μl magnetic beads (New England Biolabs [NEB]; goat anti-rabbit magnetic beads; cat. no. S1432S) was added, and samples were washed three times and rotated for 2 h at 4°C. The beads were washed six times and incubated with 300 μl elution buffer (5 mM Tris-HCl [pH 7.5], 1 mM EDTA [pH 8.0], 0.05% SDS, and 8.4 μl 10-mg/ml proteinase K) for 1.5 h at 50°C. The RNA was purified using phenol-chloroform and precipitated with ethanol. For qRT-PCR, cDNA was synthesized using a reverse transcriptase mix (Vazyme), and relative quantification was performed using specific primers. The data were normalized to the quantification cycle (*C_q_*) values of *GAPDH*. For Northern blotting, the purified RNA was run on a 1.0% agarose gel containing 2.2 M formaldehyde for 11 h at 35 V, followed by transfer to a Hybond-N+ membrane and UV cross-linking. Finally, membranes were hybridized with a DIG-labeled SARS-CoV-2 probe (nt 28274 to 29870), and probe detection was performed using a luminescence detection kit II (Roche) according to the manufacturer’s protocol. Signals were detected using a ChemiDoc MP imaging system (Tanon 5200).

### Sumoylation and ubiquitination assays.

Sumoylation and ubiquitination assays were performed as previously described (71). Briefly, the indicated plasmids were cotransfected into HEK293T cells, and the cell lysates were harvested by centrifugation at 16,000 × *g* at 4°C for 10 min. Next, 50 μl protein G Dynabeads was incubated with 10 μg of the indicated antibodies for 2 h, followed by incubation with cell lysates overnight. The complexes were washed six times with PBS containing 0.02% Tween 20 and subjected to Western blotting.

### Statistical analysis.

The statistical analysis of the qRT-PCR data was performed using two-tail unpaired *t* tests in Prism Software (GraphPad Software, La Jolla, CA, USA.). Data are presented as means ± standard deviations (*n* = 3). All experiments were repeated at least three times.
