# Supplementary figures and images for: Methyltransferase-like 3 Modulates Severe Acute Respiratory Syndrome Coronavirus-2 RNA N6-Methyladenosine Modification and Replication
Source: mBio. 2021 Jul 6;12(4):e01067-21. doi: 10.1128/mBio.01067-21 (PMC8437041; doi:10.1128/mBio.01067-21)

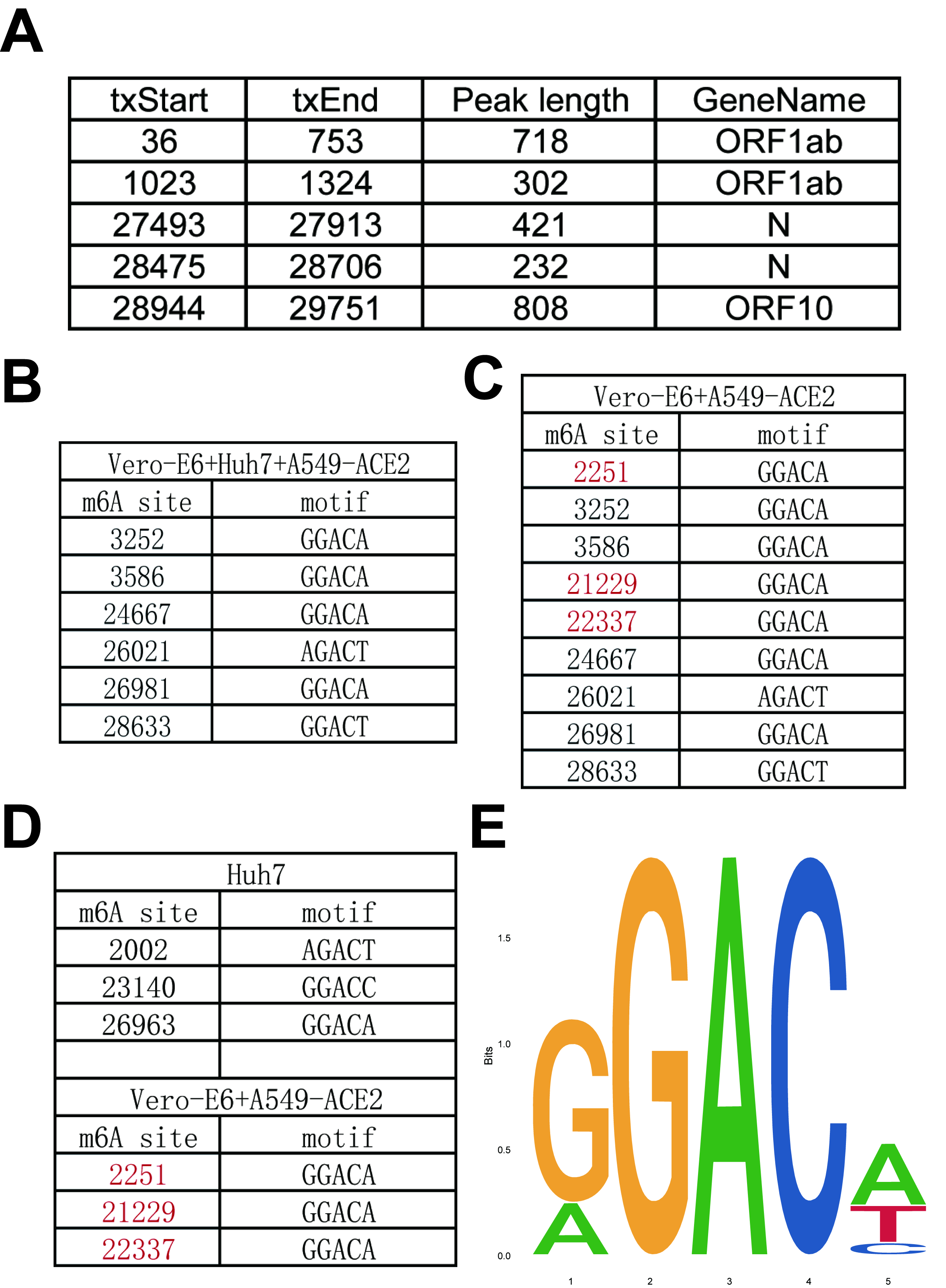

Supplement: FIG S1 [file mbio.01067-21-sf001.tif]

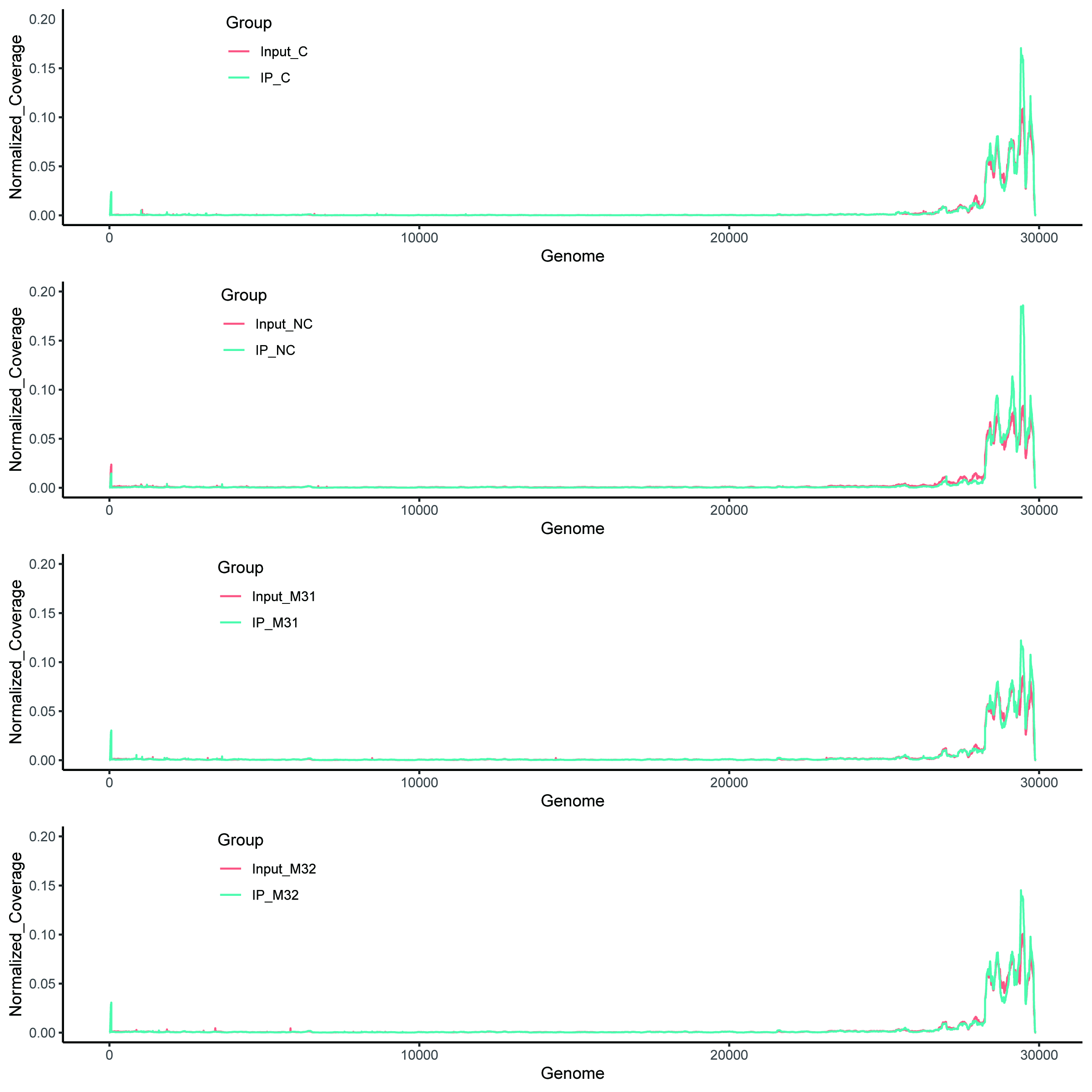

Supplement: FIG S6 [file mbio.01067-21-sf006.tif]

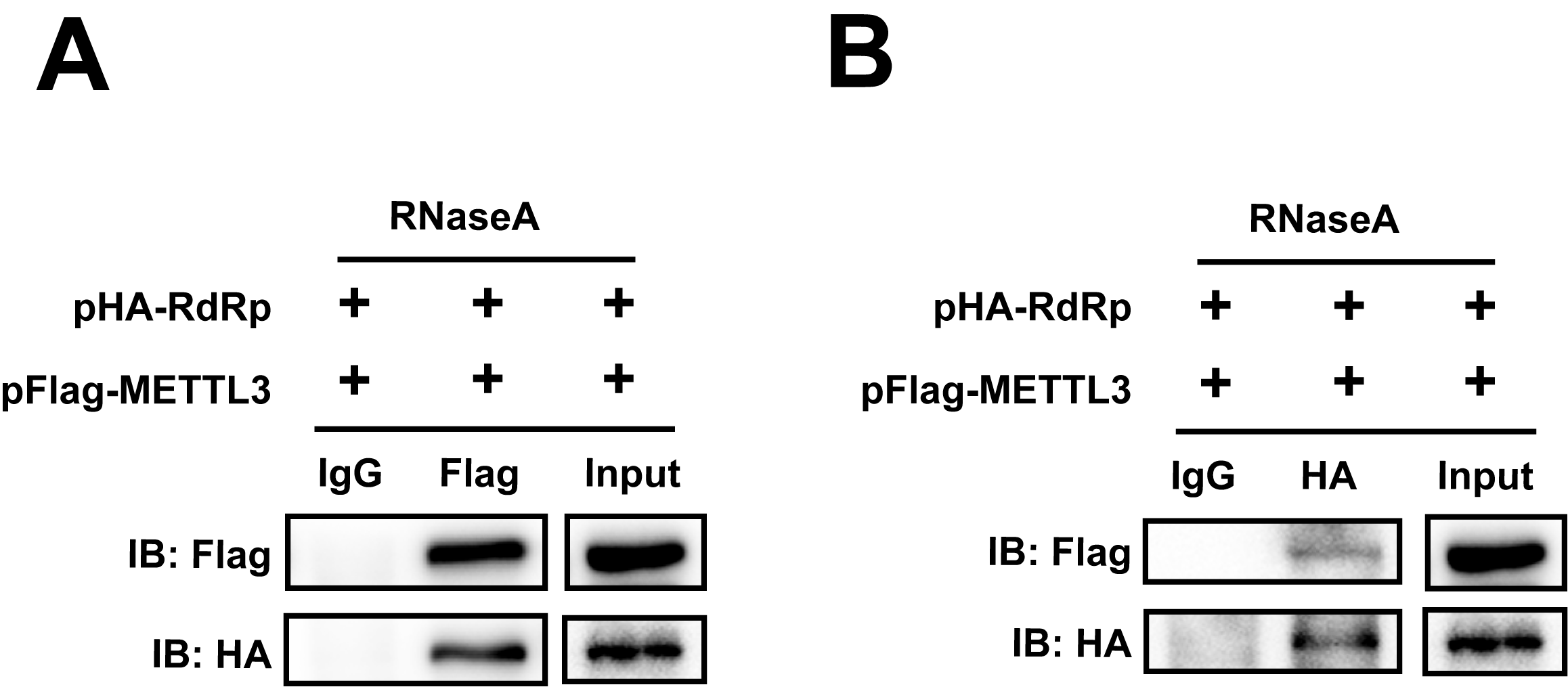

Supplement: FIG S2 [file mbio.01067-21-sf002.tif]

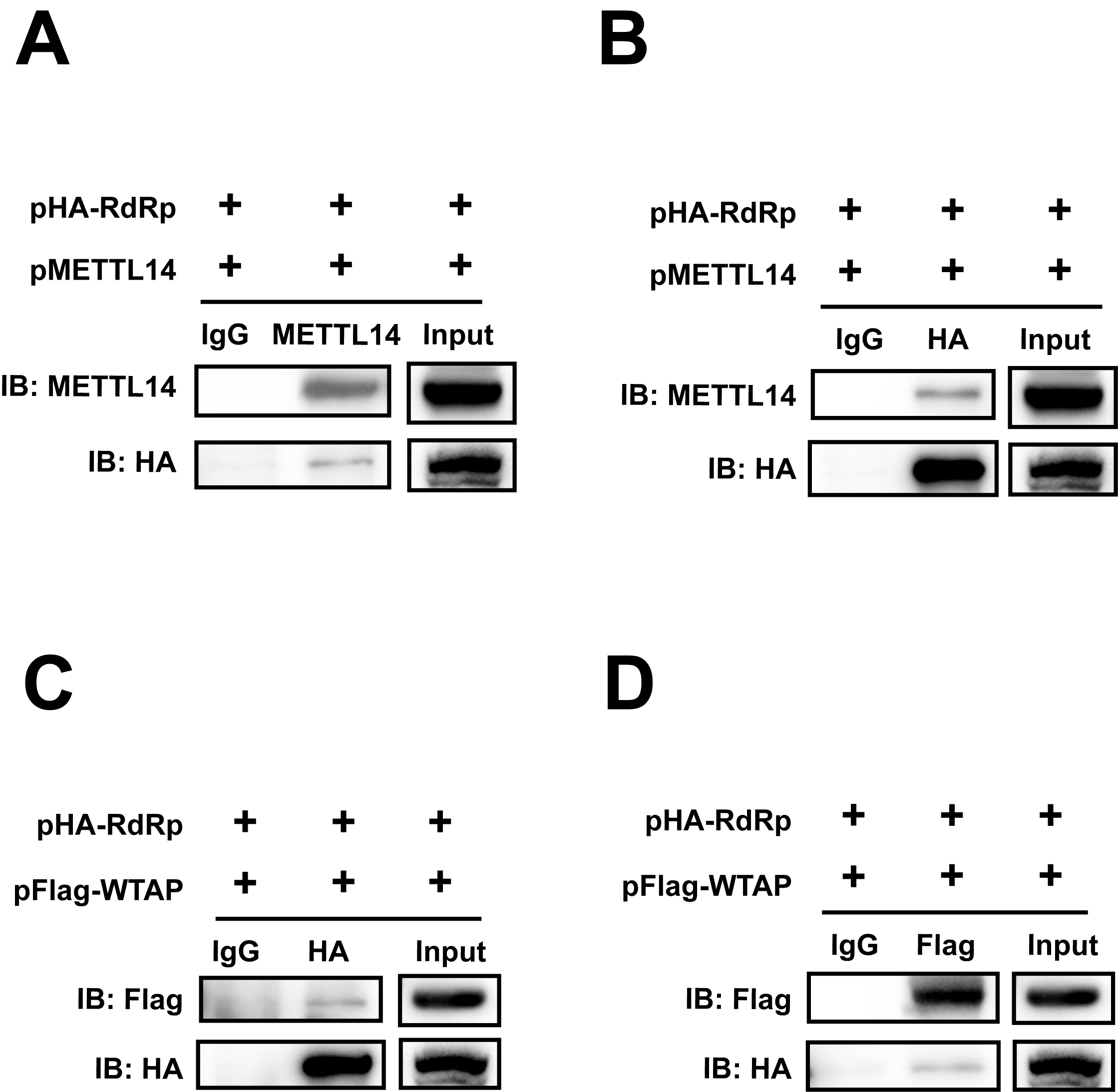

Supplement: FIG S4 [file mbio.01067-21-sf004.tif]

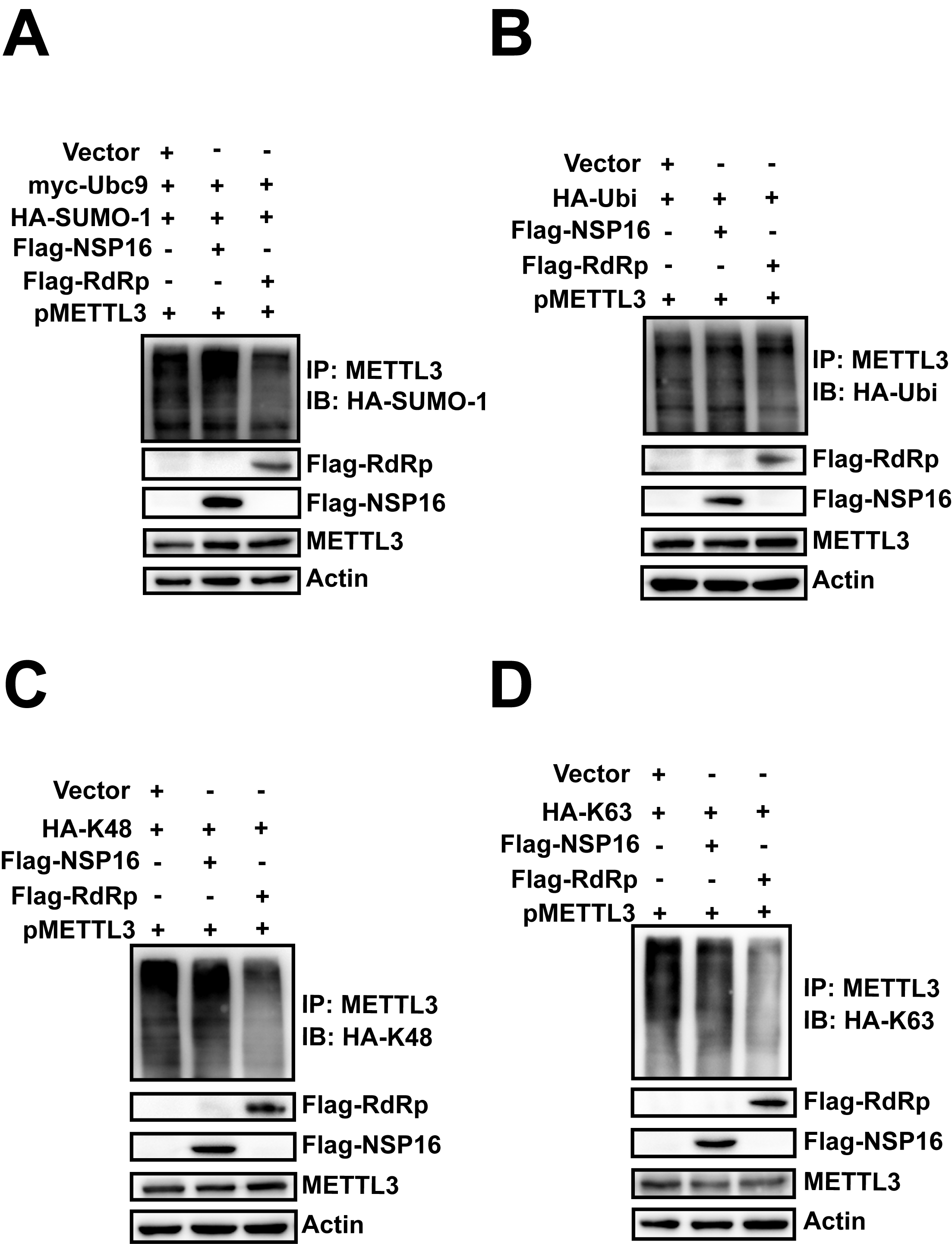

Supplement: FIG S3 [file mbio.01067-21-sf003.tif]

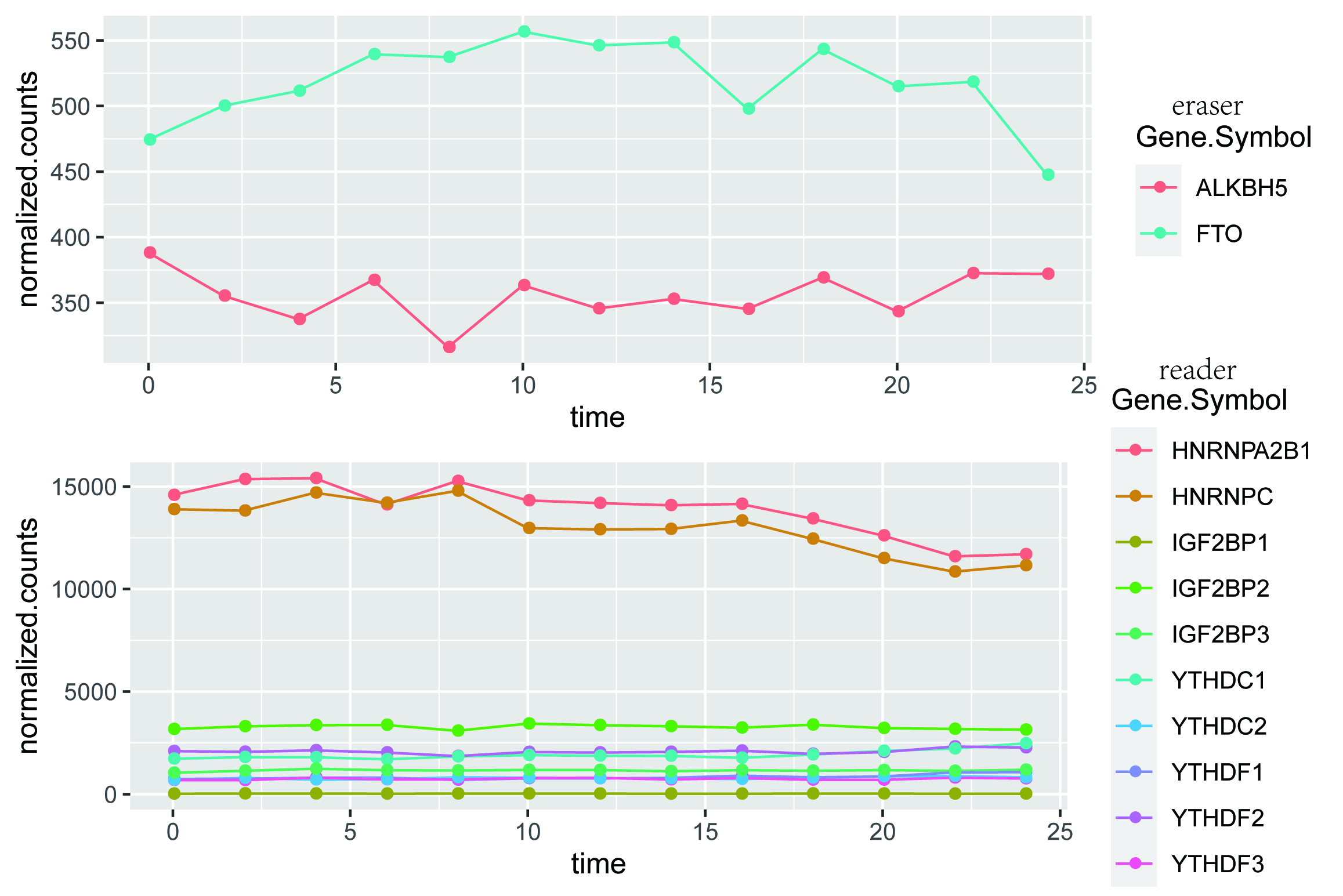

Supplement: FIG S5 [file mbio.01067-21-sf005.tif]
