# Supplementary material for: Methyltransferase-like 3 Modulates Severe Acute Respiratory Syndrome Coronavirus-2 RNA N6-Methyladenosine Modification and Replication
Source: mBio. 2021 Jul 6;12(4):e01067-21. doi: 10.1128/mBio.01067-21 (PMC8437041; doi:10.1128/mBio.01067-21)
Supplement: TABLE S1 [file mbio.01067-21-st001.docx]

| **Table S1 GAPDH, N and RdRp gene specific primers** | |
| --- | --- |
| CorV-N-QF | TAACCAGAATGGAGAACGCAGTG |
| CorV-N-QP | ATCAAAACAACGTCGGCCCCAAGGT |
| CorV-N-QR | TGAGTGAGAGCGGTGAACCAAGAC |
|  |  |
| GAPDH-qF | CAAGGGCATCCTGGGCTACACT |
| GAPDH-qP | TCTCCTCTGACTTCAACAGCGACACCCAC |
| GAPDH-qR | CCCAGCGTCAAAGGTGGAGGA |
|  |  |
| CorV-RdRp-QF | CAAAATGYTGGACTGAGACTGACC |
| CorV-RdRp-QR | ACGATATCATCDACAAAACAGCCG |
| CorV-RdRp-QP | ATCTGGGTAAGGMAGGTACACRTAATCATCAC |
|  |  |
